# Does just fungal evidence increase the mortality of critically ill patients with liver cirrhosis? A retrospective analysis

**DOI:** 10.1186/cc12344

**Published:** 2013-03-19

**Authors:** T Lahmer, U Mayr, M Messer, B Saugel, C Schultheiss, R Schmid, W Huber

**Affiliations:** 1 University Hospital Munich, Germany

## Introduction

Patients with liver cirrhosis (LC) are susceptible to a variety of complications. Especially, infection disorders can reduce their life expectancy markedly. The aim of this retrospective analysis was to detect the incidence and prognosis in patients with LC and fungal evidence (FE).

## Methods

A 3-year retrospective analysis of ICU patients with LC. Patients with LC with/without FE were compared with a matched control group based on comparable age and APACHE II score. Specimens were collected based on clinical and/or laboratory (infection signs) reasons and were analyzed by microbiological standard methods.

## Results

A total of 101 patients with LC were enrolled in this analysis. Basic data for both groups: Child-Pugh Score: 4*A, 18*B, 79*C; reason for ICU admission: 28*hepatorenal syndrome, 18*SBP, 20*GI bleeding, 19*sepsis, 16*pneumonia; etiology of LC: 83*alcoholic, 8*NASH, 8*viral (hepatitis B/C), 2*PSC. Fifty-five (54%) patients with LC had FE (mean age: 57.6 ± 14 years, sex M/F: 40/15, APACHE II score 27 ± 2). Compared with the 46 patients with LC but without FE (mean age: 59.2 ± 12 years, sex M/F: 35/11, APACHE II score 23 ± 4), these patients have a significantly higher APACHE II score (27 ± 2 vs. 23 ± 4; *P *0.001) and significantly higher mortality (43/55 (78%) vs. 15/46 (33%); *P *0.001). Multivariate analysis showed that FE is associated with a higher mortality (*P *0.001) but is not associated with age, sex or CHILD score. Seventeen (31%) of the 55 patients in the control group had FE (screening patients *n *= 147, mean age 65.8 ± 12 years, sex M/F: 35/20, APACHE II score 26.5 ± 3, reason for ICU admission: 19*pneumonia, 16*sepsis, 9*intracerebral haemorrhage, 7*acute abdomen, 4*lymphatic disorder). The mortality rate was significantly lower (27/55 (49%) vs. 43/55 (78%); *P *= 0.003) as compared with patients with LC and FE. The main localisation of fungal was in both groups the lung (46 vs. 14), followed by urine (18 vs. 2), blood culture (6 vs. 1) and ascites (4 vs. 0). Predominantly candida species were detected (especially *C. albicans/glabrata*), five patients in the LC group had aspergillus fumigatus. In LC patients, FE could be detected in many cases in several compartments.

## Conclusion

ICU patients with LC have a high incidence of FE. The mortality rate is therefore significantly higher as compared with patients with LC without FE and also as compared with a control group without LC. Therefore early antimycotic treatment should be considered.

